# CircUCK2 promotes hepatocellular carcinoma development by upregulating UCK2 in a mir-149-5p-dependent manner

**DOI:** 10.1007/s12672-024-00863-y

**Published:** 2024-01-20

**Authors:** Minghai Shen, Qinghua Zhang, Wanneng Pan, Bei Wang

**Affiliations:** 1 Department of General Surgury, Xixi Hospital of Hangzhou, Hangzhou, 310023 China; 2 Department of Hepatobiliary and Pancreatic Surgery, First Affiliated Hospital, Zhejiang University School of Medicine, No. 79, Qingchun Road, Shangcheng District, Hangzhou, 310023 China

**Keywords:** HCC, circUCK2, miR-149-5p, UCK2

## Abstract

**Background:**

Circular RNAs (circRNAs) participate in the regulation of Hepatocellular Carcinoma (HCC) progression. The objective of this study was to explore the function and mechanism of circUCK2 in HCC development.

**Methods:**

The RNA levels of circUCK2, miR-149-5p and uridine–cytidine kinase 2 (UCK2) were examined by quantitative real-time polymerase chain reaction (qRT-PCR). EdU incorporation assay and colony formation assay were respectively performed to analyze cell proliferation and colony formation. Wound healing assay and transwell assay were conducted for cell migration and invasion. Flow cytometry was used for cell apoptosis analysis. Western blot assay was conducted to determine the protein levels of E-cadherin, N-cadherin, matrix metallopeptidase 9 (MMP-9) and UCK2. Dual-luciferase reporter assay, RNA immunoprecipitation (RIP) assay and RNA pull-down assay were conducted to confirm the interaction between miR-149-5p and circUCK2 or UCK2. The xenograft model was established to explore the role of circUCK2 in tumor growth in vivo.

**Results:**

CircUCK2 level was elevated in HCC, and circUCK2 depletion suppressed HCC cell proliferation, colony formation, migration and invasion and accelerated cell apoptosis. Mechanistically, circUCK2 could positively modulate UCK2 expression by interacting with miR-149-5p. Furthermore, the repressive effects of circUCK2 knockdown on the malignant behaviors of HCC cells were alleviated by UCK2 overexpression or miR-149-5p inhibition. The promoting effects of circUCK2 overexpression on HCC cell malignancy were alleviated by UCK2 silencing or miR-149-5p introduction. Additionally, circUCK2 knockdown hampered tumor growth in vivo.

**Conclusion:**

CircUCK2 contributed to HCC malignant progression in vitro and in vivo via targeting miR-149-5p/UCK2 axis, demonstrating that circUCK2 might be a novel therapeutic target for HCC.

**Supplementary Information:**

The online version contains supplementary material available at 10.1007/s12672-024-00863-y.

## Introduction

Encountered in the context of chronic liver diseases, hepatocellular carcinoma (HCC) has high morbidity [1, 2]. Although significant improvements have been gained on HCC treatment, the advanced and metastatic HCC is harder to cure [3]. Hence, it is imperative to explore molecular basis responsible for HCC pathogenesis and seek better curative strategies for HCC patients.

Circular RNAs (circRNAs) are circular non-coding RNAs (ncRNAs) that are highly abundant in eukaryotes [4]. Increasing evidence illuminates that circRNAs exert a pivotal role in diversiform cancers [5–7], including HCC [8]. For example, circ_101555 contributed to HCC cell growth and transferability by segregating miR-145-5p from CDCA3 [9]. Circ_0005397 expedited the advancement of HCC by modulating miR-326/PDK2 axis [10]. CircUCK2 (hsa_circ_0006758), derived from uridine–cytidine kinase 2 (UCK2) mRNA, is a circRNA with largely unknown function. According to the results of high-throughput sequencing, circUCK2 had elevated expression in HCC tissues [11]. Nevertheless, the precise role of circUCK2 in HCC malignant progression is unknown.

MicroRNAs (miRNAs) post-transcriptionally control gene expression via recognizing the 3′UTRs of target mRNAs [12, 13]. Various miRNAs are crucial mediators in the tumorigenesis of HCC through promoting or suppressing tumors [14]. MiR-149-5p participated in HCC development [15]. For instance, it had a decreased level in HCC and was related to the proliferative capacity and transferability of HCC cells [16]. UCK2, the precursor mRNA of circUCK2, has been claimed to be overexpressed and connected with the aggressiveness and poor clinical outcomes of HCC [17]. In our preliminary experiments, UCK2 was a possible target gene of miR-149-5p. Nonetheless, whether the biological action of circUCK2 in modulating HCC progression is mediated by miR-149-5p and UCK2 is still uninvestigated.

Here, the action of circUCK2 in HCC cell progression was analyzed. Moreover, the potential regulatory mechanism of circUCK2 related to miR-149-5p and UCK2 in hepatocellular carcinoma malignant progression was investigated, which might provide additional strategies for HCC therapy.

## Materials and methods

### Clinical tissues and serum samples

40 HCC patients and 33 healthy volunteers were recruited from Xixi Hospital of Hangzhou. 40 paired HCC tumor tissues and matched neighboring healthy tissues were collected from HCC patients. Serum samples were obtained from 33 healthy volunteers and 40 HCC patients before surgical resection. No patients had received anti-tumor therapy beforehand. The informed consent was signed by all participators. This study was proceeded after getting the approval of the Ethics Committee of the Xixi Hospital of Hangzhou. All methods in the study were carried out in accordance with the Helsinki guidelines and declaration or any other relevant guidelines.

### Cell culture

Human HCC cell lines (Huh7, HCCLM6, MHCC97-L and MHCC97-H, Cobioer Biosciences, Nanjing, China) and THLE-2 (Bluefbio, Shanghai, China) were maintained in DMEM medium (Gibco, Carlsbad, CA, USA) plus 10% FBS (Gibco) and 1% penicillin–streptomycin mixture (Solarbio, Beijing, China) in a moist circumstance under 37 °C with 5% CO_2_.

### Cell transfection

To silence circUCK2 or UCK2, circUCK2 shRNAs (sh-circUCK2-1, sh-circUCK2-2 as well as sh-circUCK2-3) or UCK2 short hairpin RNA (sh-UCK2) were established by Ribobio (Guangzhou, China). The overexpression vectors of UCK2 (pcDNA-UCK2) and circUCK2 were separately constructed by Ribobio by introducing UCK2 or circUCK2 sequence into pcDNA3.1 vector or pCD5-ciR, with pcDNA and vector served as negative controls, respectively. MiR-149-5p mimic or inhibitor (miR-149-5p or anti-miR-149-5p) was constructed by Ribobio, with miR-NC or anti-NC as matched negative control. MHCC97-H and HCCLM6 cell transfection was performed through Lipofectamine 3000 (Invitrogen, Carlsbad, CA, USA).

### Quantitative real-time PCR (qRT-PCR)

As instructed by the guidebook of Trizol reagent (Invitrogen), RNA segregation assays were executed. The synthesis of cDNA was acquired from RNA utilizing a SuperRT Reagent Kit (CWBIO, Beijing, China) or miRNA RT-PCR reagents (Yeasen, Shanghai, China). Subsequently, qRT-PCR assay was carried out by using an qPCR SYBR Green Master Mix (Yeasen). The 2^−ΔΔCt^ method was utilized to calculate circUCK2, miR-149-5p and UCK2 expression. Primers were exhibited in Table 1.

**Table 1:** Primer sequences used for qRT-PCR

Primers for PCR (5′-3′)		
circUCK2	Forward	TTGTCTCCCATTCCCGTCTTC
	Reverse	ACGGTAGAAGCTATCCTGGC
miR-149-5p	Forward	TCTGGCTCCGTGTCTTC
	Reverse	GAACATGTCTGCGTATCTC
UCK2	Forward	CGGCTCTCACGCAGAGTATT
	Reverse	CAAAGGCAGGCTTGACGAAC
GAPDH	Forward	GTCTCCTCTGACTTCAACAGCG
	Reverse	ACCACCCTGTTGCTGTAGCCAA
U6	Forward	CTCGCTTCGGCAGCACA
	Reverse	AACGCTTCACGAATTTGCGT

### RNase R assay

Two µg isolated RNA from MHCC97-H and HCCLM6 cells was digested utilizing RNase R (Lucigen Corporation, Middleton, WI, USA) to identify the stabilization of circUCK2,. Then, relative RNA abundances of circUCK2 and counterpart UCK2 were assessed by qRT-PCR analysis.

### Subcellular fractionation location assay

The mirVana PARIS Kit (Ambion, Austin, TX, USA) was applied to separate nucleocytoplasmic RNAs from MHCC97-H and HCCLM6 cells as instructed by the guidebook. Subsequently, circUCK2 expression in nucleus and cytoplasm was detected via qRT-PCR.

### 5-Ethynyl-2′-deoxyuridine (EdU) incorporation assay

Cells were plated in 96-well plates, trained and immobilized with 4% paraformaldehyde (Solarbio) prior to permeabilization with 0.5% Triton X-100 (Solarbio). Whereafter, cells were exposed to EdU and DAPI solution (Solarbio). At last, fluorescence microscope (100×; Olympus, Tokyo, Japan) was employed to count EdU-positive cells.

### Colony formation assay

MHCC97-H and HCCLM6 cells were plated in 6-well plates (about 500 cells/well), and paraformaldehyde (Solarbio) and crystal violet (Solarbio) were continuously conducted to immobilize and dye the colonies for 30 min, severally, after 2-week culture. The images were photographed, and generated colonies (> 50 cells) was counted using microscope (Olympus).

### Flow cytometry

MHCC97-H and HCCLM6 cells were harvested and resuspended in 200 µL binding buffer after washing. Subsequently, cells were dyed with Annexin V-FITC for 10 min and propidium iodide (PI) for 15 min. Apoptotic cells were quantified using flow cytometer.

### Wound healing assay

To assess the migratory capacity of MHCC97-H and HCCLM6 cells, wound healing assay was implemented. Transfected cells were subjected to 24-hour incubation in 6-well plates and gently scratched using 200 µL pipette tips (sterile). Later, cells were exposed to serum-free medium. At 0 and 24 h, the widths of wounds were recorded and photographed with a microscope (Olympus; 40×).

### Transwell assay

For investigating cell invasive ability, the transduced MHCC97-H and HCCLM6 cells (1 × 10^5^) diluted in serum-free medium were placed in the top compartment of Matrigel-coated transwell chamber. 24 h later, the migrated cells were fixed, captured and counted under a microscope with 4 random areas following dyeing with 0.1% crystal violet (Solarbio).

### Western blot

The protein (20 µg per lane) prepreaed using lysis buffer (Beyotime, Shanghai, China) was added onto SDS-PAGE gels, followed by transferring onto PVDF membrane (Beyotime). Afterwards, these membranes were reacted with primary antibodies against E-cadherin (1:2000; ab133597; Abcam, Cambridge, UK), N-cadherin (1:1000; ab207608; Abcam) and matrix metallopeptidase 9 (MMP-9; 1:2000; ab76003; Abcam), UCK2 (1:1000; FNab09223; Fine Biotech Co., Ltd., Wuhan, China) or β-actin (1:200; ab115777; Abcam). Protein blots were tested by BeyoECL reagents (Beyotime).

### Immunohistochemistry (IHC) assay

As instructed by the reported method [18], paraffin-embedded tissue sections were co-reacted with primary UCK2 antibody (1:200; FNab09223; Fine Biotech Co., Ltd.) overnight at 4 °C. Afterward, these sections were exposed to DAB (Beyotime) as well as hematoxylin (Beyotime) in sequence. At last, representative images were acquired using a fluorescence microscope (Olympus).

### RNA immunoprecipitation (RIP) assay

As instructed by the guidebook of EZ-Magna RIP Kit (Millipore, Billerica, MA, USA), we peroformed RIP assay. Briefly, MHCC97-H and HCCLM6 cells (1 × 10^7^) were exposed to RIP lysis buffer and the lysates were then reacted with magnetic beads combined with antibody against anti-IgG or anti-Ago2 at 4 °C. qRT-PCR analysis was used to detect circUCK2, miR-149-5p and UCK2 contents.

### Dual-luciferase reporter assay

Potential target miRNAs of circUCK2 were analyzed using bioinformatics tools Starbase and circinteractome. The potential mRNAs binding to UCK2 were forecasted using bioinformatics tool TargetScan. The wild-type fragment of circUCK2 or UCK2 3′UTR with putative miR-149-5p binding sequence, and the mutant fragment of circUCK2 or UCK2 3′UTR containing mutated miR-149-5p matched sites were separately amplified and introduced into pGL3-basic vector (Promega, Madison, WI, USA), forming the fusion plasmids WT-circUCK2, WT-UCK2 3′UTR, MUT-circUCK2 and MUT-UCK2 3′UTR. Next, the constructed plasmids were transduced into MHCC97-H and HCCLM6 cells with miR-149-5p or miR-NC by Lipofectamine 3000 (Invitrogen). Luciferase intensity was determined after 48 h-transfection by exploiting the Dual-Lucy Assay Kit (Solarbio).

### RNA pull-down assay

Biotin-miR-149-5p-WT and biotin-miR-149-5p-MUT or control (biotin-NC) were introduced into MHCC97-H and HCCLM6 cells. The harvested cells were dissociated utilizing RIPA buffer. Then the cell lysates were interacted with magnetic beads (Invitrogen) at 4 °C. qRT-PCR assay was performed for the enrichment levels of circUCK2 and UCK2.

### Xenograft tumor model

BALB/c nude mice (male, 4–6 weeks old, Hunan Slyke Jingda Experimental Animal Co., LTD, Changsha, China) were randomly divided into two groups (*n* = 5/group): sh-circUCK2 or sh-NC group. Then HCCLM6 cells (2 × 10^6^) with sh-circUCK2 or sh-NC transfection were re-suspended in 0.2 mL PBS (Solarbio) and then inoculated into the flanks of the mice through subcutaneous injection. The tumor volume was measured every 7 days, and calculated via the formula: length × width^2^/2 [19]. At the 35th day post-injection, mice were sacrificed using 5% isoflurane, and the transplanted neoplasms were harvested for tumor weight and subsequent experiments. The study about animal experiment got authorization from the Animal Care and Use Committee of the Xixi Hospital of Hangzhou and performed in accordance with the guidelines of the National Animal Care and Ethics Institution. It was carried out in compliance with the ARRIVE guidelines.

### Statistical analysis

All data from at least 3 repeats were processed using GraphPad Prism 7 Software and presented as mean ± standard deviation. The comparisons of differences were conducted utilizing Student’s *t*-test, or one-way analysis of variance. It was deemed to be statically significant when *P* < 0.05.

## Results

### CircUCK2 was overexpressed in HCC tissues

**Fig. 1:**
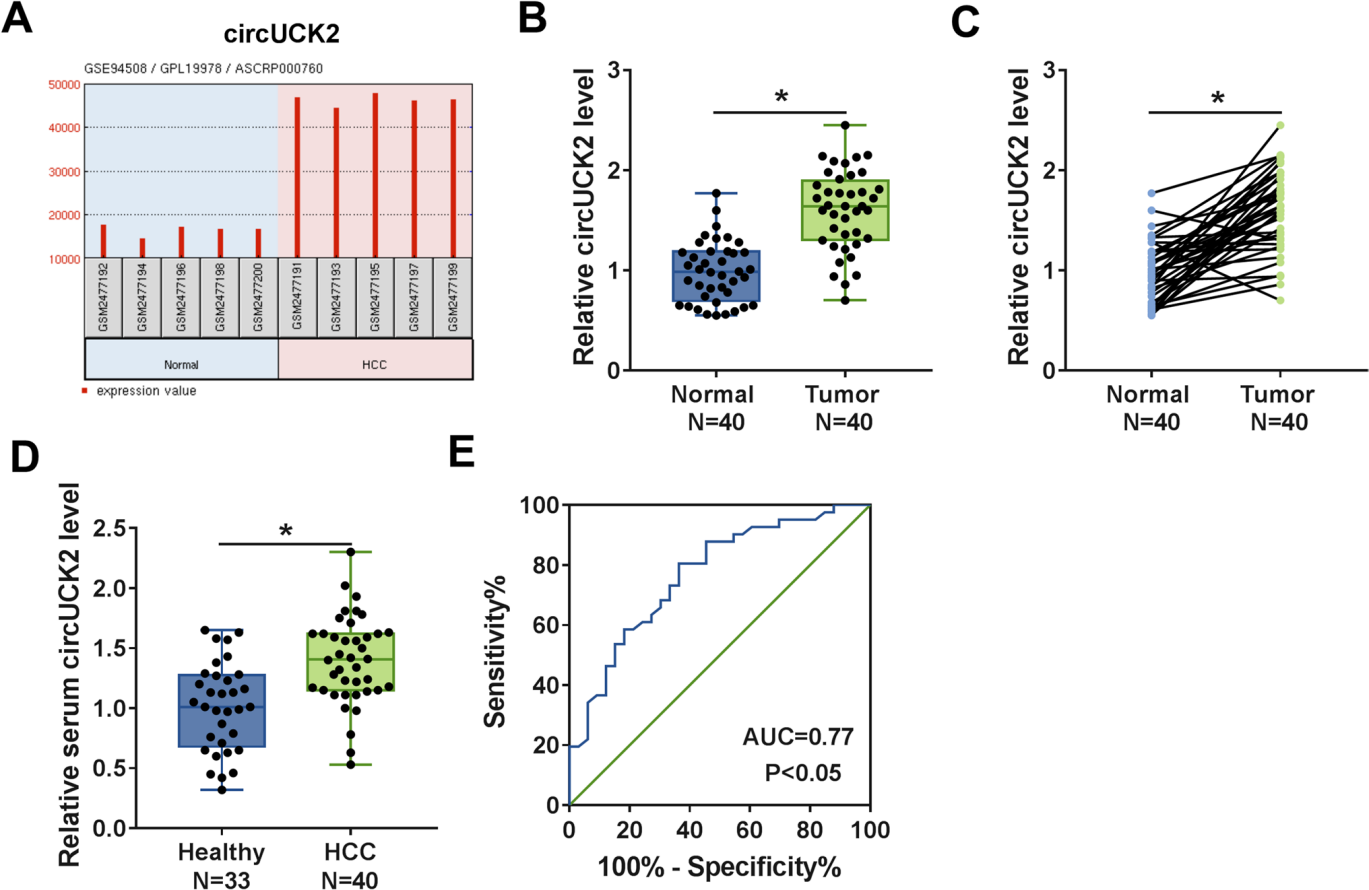
CircUCK2 was upregulated in HCC tissues and serum. **A** Gene Expression Omnibus (GEO) database exhibited the expression of circUCK2 in 5 pairs of HCC tumor tissues (HCC) and adjacent normal tissue (Normal) according to GSE94508 dataset. **B**, **C** The expression of circUCK2 in 40 paired HCC tissues and adjacent normal tissues was detected by qRT-PCR assay. **D** The level of circUCK2 in serum samples of 33 healthy participators and 40 HCC patients was examined by qRT-PCR assay. **E** ROC curve presented the clinical significance of circUCK2 in HCC. *P < 0.05

According to GSE94508 dataset, the expression of circUCK2 was higher in HCC tumor tissues (*N* = 5) than in matched adjacent normal tissues (*N* = 5) (Fig. 1A). To explore the biological role of circUCK2 in HCC progression, the expression of circUCK2 was detected in HCC tissues and cells. In accordance with GSE94508 dataset, in this study, qRT-PCR assay illustrated that circUCK2 expression was strikingly enhanced in HCC tissues (*N* = 40) compared to that in adjacent normal tissues (*N* = 40) (Fig. 1B, C). Similarly, the level of circUCK2 was also increased in the serum samples (*N* = 40) from HCC patients relative to that in normal serum samples (*N* = 33) from healthy volunteers (Fig. 1D). To evaluate the efficiency of circUCK2 as a diagnostic biomarker for HCC, the receiver operating characteristic (ROC) curve analysis was conducted. ROC curve results exhibited that the area under the ROC curve (AUC) was 0.77 (Fig. 1E), suggesting that circUCK2 might have diagnostic value for HCC patients. These data indicated that high expression of circUCK2 might be associated with the malignant development of HCC.

### CircUCK2 was a circular and stable transcript in HCC

**Fig. 2:**
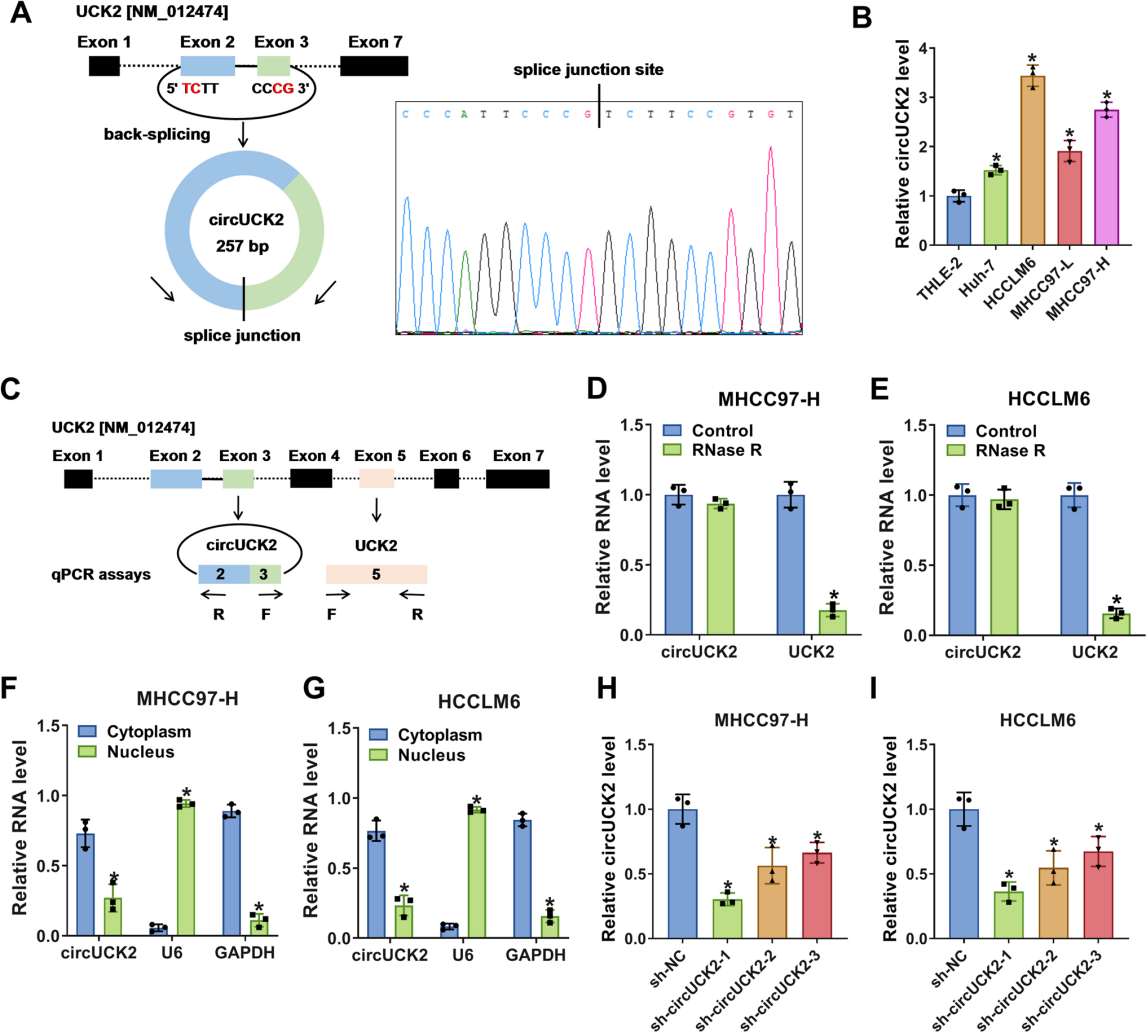
CircUCK2 was a circular and stable transcript in HCC. **A** The formation of circUCK2 was shown and the splice junction site of circUCK2 was validated through sequencing of its qRT-PCR product. **B** The level of circUCK2 in HCC cell lines (Huh7, HCCLM6, MHCC97-L and MHCC97-H) and normal THLE-2 cells was measured via qRT-PCR assay. **C** The schematic illustration showed the mechanism of designing circUCK2 and UCK2 primers. **D**, **E** The levels of circUCK2 and linear UCK2 mRNA in MHCC97-H and HCCLM6 cells after RNase R treatment were evaluated by qRT-PCR assay. **F**, **G** The distribution of circUCK2 in the cytoplasm and nucleus of MHCC97-H and HCCLM6 cells were tested by subcellular fractionation location assay. **H**, **I** The level of circUCK2 in MHCC97-H and HCCLM6 cells introduced with sh-circUCK2 (− 1, − 2 and − 3) or sh-NC was determined by qRT-PCR assay. *P < 0.05

Next, the circular characteristics of circUCK2 were examined through circRNA sequencing. It was affirmed that circUCK2 was back-spliced from UCK2 gene, ranging from the 2nd exon to the 3rd exon of the host, which contains two exons (Fig. 2A). Sanger sequencing was used to validate head-to-tail splice junction for circUCK2 which was identical to the reported sequence in circBank (Fig. 2A). Similar to the result in tissues, the level of circUCK2 was prominently increased in human HCC cell lines (Huh7, HCCLM6, MHCC97-L and MHCC97-H) versus that in normal THLE-2 cells (Fig. 2B). For the higher expression of circUCK2 in HCC cell lines, MHCC97-H and HCCLM6 cells were selected for subsequent experiments. The mechanism of designing circUCK2 and UCK2 primers is shown in Fig. 2C. CircUCK2 was resistant to RNase R digestion in MHCC97-H and HCCLM6 cells in vitro compared with the linear transcript UCK2 mRNA (Fig. 2D, E), implying the stability of circUCK2. Besides, the subcellular fractionation location assay demonstrated that circUCK2 was mainly located in the cytoplasm but not in the nucleus of MHCC97-H and HCCLM6 cells (Fig. 2F, G), providing the possibility for the combination of circUCK2 and miRNAs. For decreasing the endogenous level of circUCK2, MHCC97-H and HCCLM6 cells were introduced with shRNA targeting circUCK2 (sh-circUCK2-1, sh-circUCK2-2 or sh-circUCK2-3). QRT-PCR assay suggested that circUCK2 level was significantly attenuated in MHCC97-H and HCCLM6 cells after transfected of sh-circUCK2 (− 1, − 2 and − 3), indicating that the transfection was successful (Fig. 2H, I). Therein, sh-circUCK2-1 (sh-circUCK2) with the highest knockdown efficiency was selected for subsequent explorations. These results manifested that circUCK2 was an upregulated circRNA with a stable structure in HCC.

### CircUCK2 knockdown suppressed proliferation, migration, invasion and promoted apoptosis in HCC cells

**Fig. 3:**
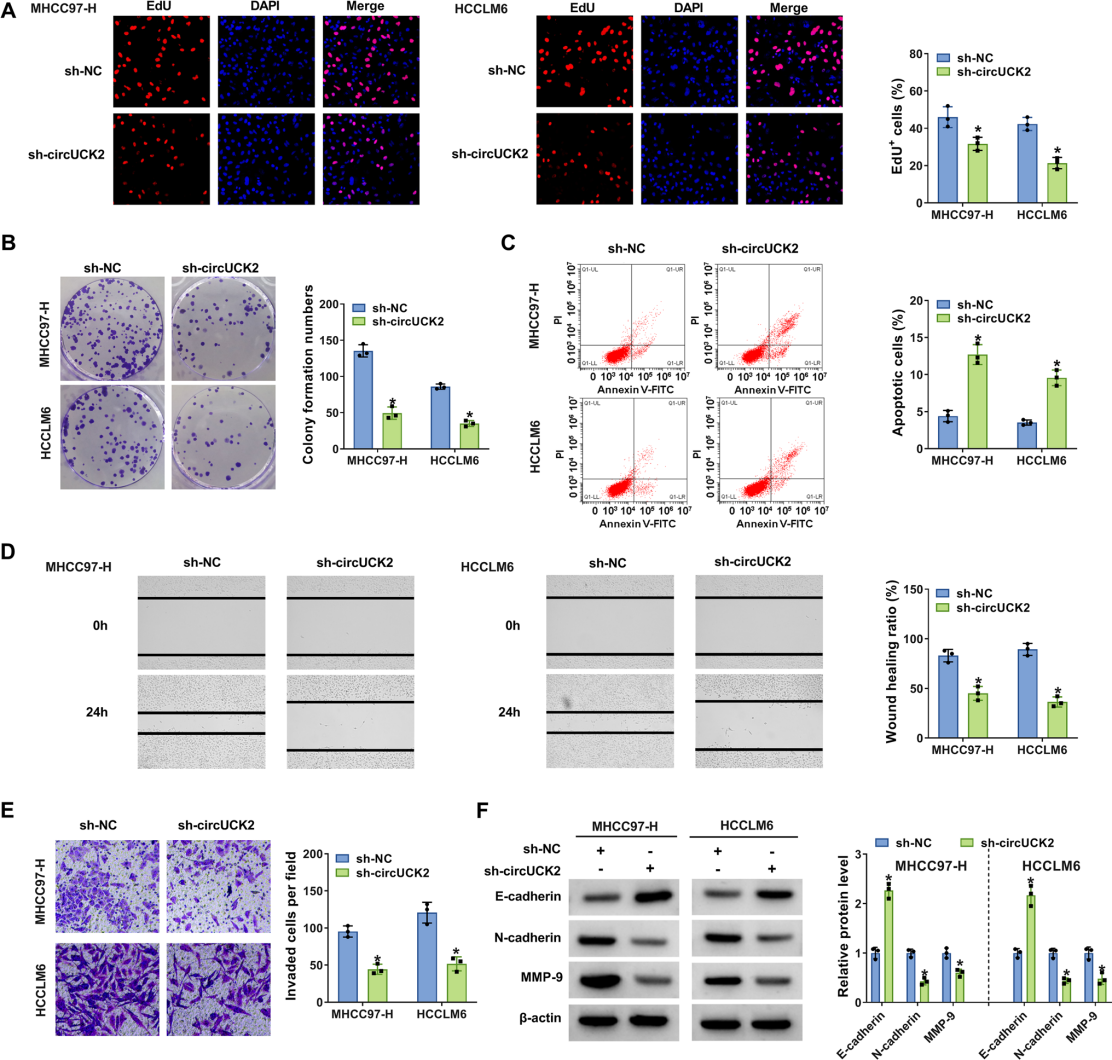
CircUCK2 depletion repressed the proliferation, migration, invasion and enhanced apoptosis in HCC cells. MHCC97-H and HCCLM6 cells were transfected with sh-circUCK2 (sh-circUCK2-1) or sh-NC. **A** Cell proliferation was assessed using the EdU incorporation assay. EdU-labeled cells existed in purple as the EdU (red) was co-localized with DAPI (blue). **B** Colony formation ability was investigated by colony formation assay. **C** Cell apoptosis was monitored by flow cytometry. **D**, **E** Cell migration and cell invasion were determined by wound healing assay and transwell assay, respectively. **F** The protein levels of E-cadherin, N-cadherin and MMP-9 in transfected MHCC97-H and HCCLM6 cells were assessed using western blot. *P < 0.05

To investigate the functional role of circUCK2, the loss-of-function assay was conducted in MHCC97-H and HCCLM6 cells transfected with sh-circUCK2 or sh-NC. EdU assay manifested that circUCK2 deficiency significantly impeded the proliferation of MHCC97-H and HCCLM6 cells, reflected by a reducing number of EdU-stained cells (Fig. 3A). Colony formation assay confirmed that the colony formation capacities of MHCC97-H and HCCLM6 cells were markedly suppressed by circUCK2 knockdown (Fig. 3B). Conversely, flow cytometry analysis demonstrated that circUCK2 deficiency strikingly promoted the apoptosis of MHCC97-H and HCCLM6 cells (Fig. 3C). Wound healing assay depicted that wound healing rates of MHCC97-H and HCCLM6 cells were distinctly depressed at the presence of sh-circUCK2 (Fig. 3D), indicating that circUCK2 depletion restrained the migratory ability of MHCC97-H and HCCLM6 cells. Transwell assay presented that circUCK2 downregulation notably lessened the number of invaded cells (Fig. 3E). Moreover, the effects of circUCK2 on epithelial-mesenchymal transition (EMT)-associated proteins (E-cadherin, N-cadherin and MMP-9) were further explored via western blot assay. The result validated that the protein level of epithelial marker (E-cadherin) was increased while the levels of mesenchymal markers (N-cadherin and MMP-9) were declined in MHCC97-H and HCCLM6 cells transfected with sh-circUCK2 (Fig. 3F). The efficiency of circUCK2 overexpresison is shown in Figure S1A. CircUCK2 introduction promoted the proliferation, migration and invasion and inhibited apoptosis of MHCC97-H cells (Figure S1D–H). As shown in Figure S1I, the increased circUCK2 expression led to promotion in UCK2, N-cadherin and MMP-9 protein expression and inhibition in E-cadherin protein expression. Taken together, circUCK2 knockdown inhibited the malignant behaviors of HCC cells.

### UCK2 was upregulated in HCC tissues and cells

**Fig. 4:**
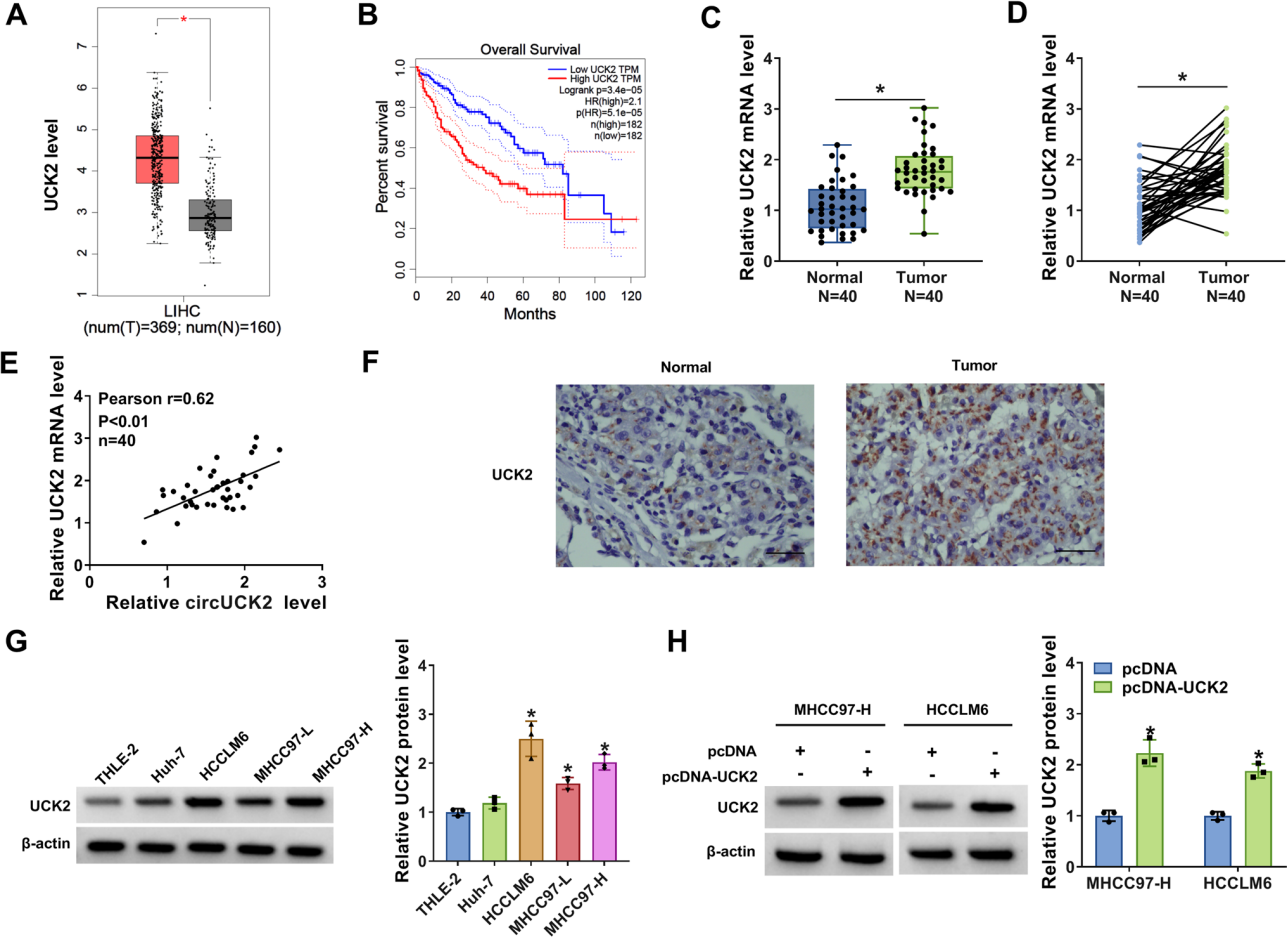
UCK2 was overexpressed in HCC tissues and cells. **A** The boxplot showed the expression of UCK2 in HCC tissues (T, *n* = 369) matched with normal tissues (N, *n* = 160) according to the GEPIA database. **B** The overall survival data of HCC patients with high expression of UCK2 (*n* = 182) and HCC patients with low expression of UCK2 (*n* = 182) was from GEPIA database. **C**, **D** The mRNA expression of UCK2 in HCC tissues (*N* = 40) and matched normal tissues (*N* = 40) was measured by qRT-PCR assay. **E** The correlation between UCK2 mRNA and circUCK2 was analyzed by Spearman’s correlation coefficient analysis. **F** The expression of UCK2 in HCC tissues and normal tissues was detected by IHC assay. **G**, **H** The protein expression of UCK2 in THLE-2, Huh7, HCCLM6, MHCC97-L and MHCC97-H cells (**G**), MHCC97-H and HCCLM6 cells transfected with pcDNA or pcDNA-UCK2 (**H**) was measured by western blot. *P < 0.05

From GEPIA database, it was monitored that the expression of UCK2 was notably increased in HCC tissues (T, *n* = 369) compared with that in normal tissues (N, *n* = 160) (Fig. 4A), and the HCC patients with the high expression of UCK2 (*n* = 182) had the lower overall survival (*P* < 0.05) than low UCK2 group (*n* = 182) based on the median value of UCK2 (Fig. 4B). Conformably, this study also confirmed that the mRNA expression of UCK2 in HCC tissues (*N* = 40) was remarkably raised relative to that in matched normal tissues (*N* = 40) (Fig. 4C, D). Furthermore, the expression of UCK2 in HCC tissues was positively correlated with circUCK2 expression (Fig. 4E). IHC assay suggested that the rate of UCK2-positive cells was elevated in HCC tissues compared to that in normal tissues (Fig. 4F), implying the enhancement of UCK2 expression in HCC tissues. Likewise, the protein expression of UCK2 in human HCC cells (HCCLM6, MHCC97-L and MHCC97-H) was enhanced relative to that in normal THLE-2 cells (Fig. 4G). As shown in Figure S2A–D, the mRNA and protein expression of UCK2 were inhibited after transfection with sh-circUCK2-1 in both MHCC97-H and HCCLM6 cell lines and transfection with sh-circUCK2-2 in HCCLM6 cells, but were not affected by sh-circUCK2-3. Then, the overexpression vector of UCK2 (pcDNA-UCK2) was constructed to study its function in HCC. The elevated expression of UCK2 in MHCC97-H and HCCLM6 cells after pcDNA-UCK2 transfection was raised, suggesting the successful transfection of pcDNA-UCK2 (Fig. 4H). Thus, UCK2 was upregulated in HCC cells and tumors.

### Overexpression of UCK2 reversed the impacts of circUCK2 knockdown on the malignant phenotypes of HCC cells

**Fig. 5:**
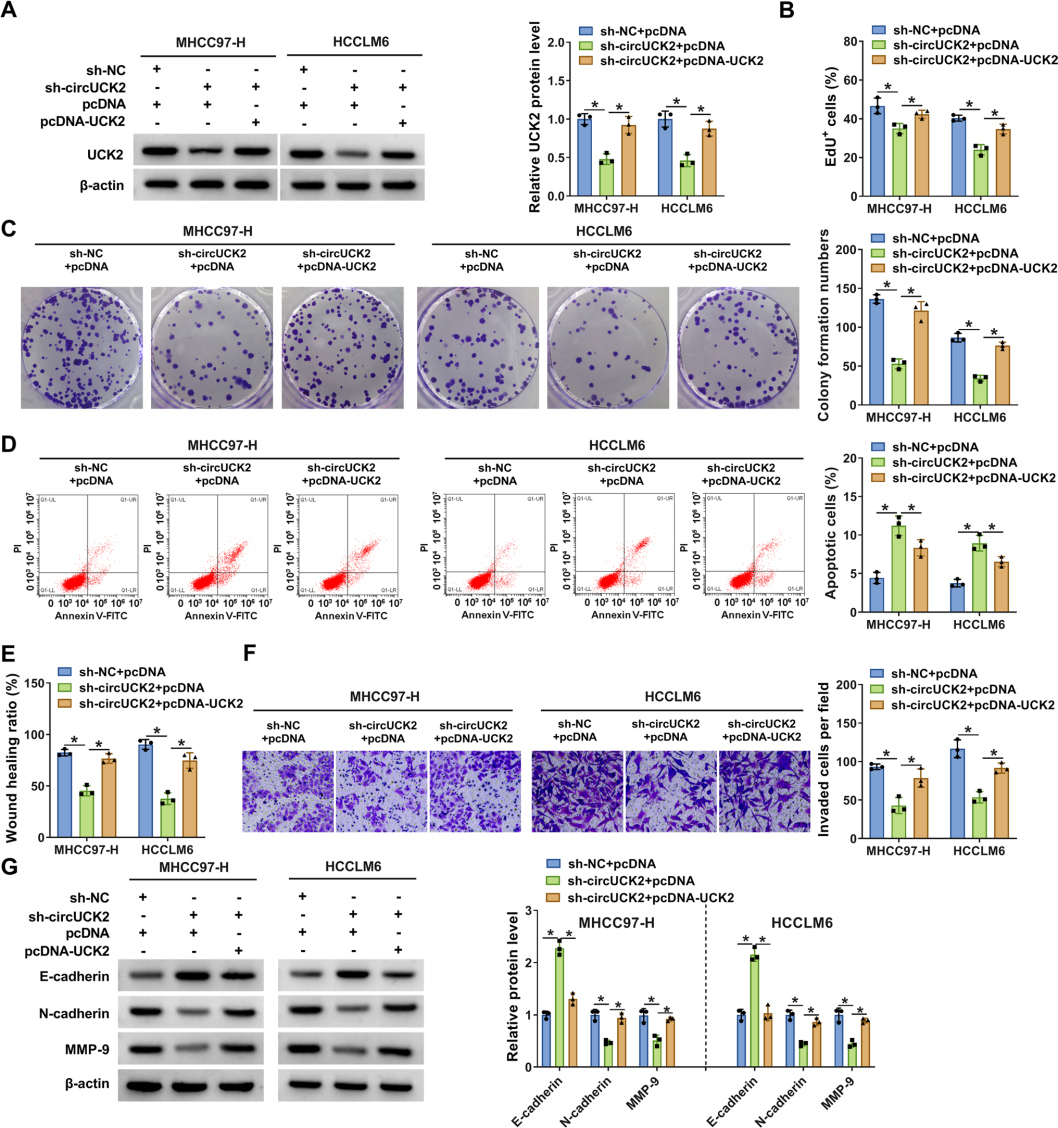
Overexpression of UCK2 relieved the influences of circUCK2 knockdown on the progression of HCC cells. MHCC97-H and HCCLM6 cells were introduced with sh-NC + pcDNA, sh-circUCK2 + pcDNA or sh-circUCK2 + pcDNA-UCK2. **A** The protein level of UCK2 was detected by western blot. Cell proliferation (**B**), colony formation (**C**), apoptosis (**D**), migration (**E**) and invasion (**F**) in transfected cells were examined by EdU incorporation assay, colony formation assay, flow cytometry, wound healing assay and transwell assay, respectively. **G** The protein levels of E-cadherin, N-cadherin and MMP-9 were measured by western blot. *P < 0.05

Considering the same varying tendencies of UCK2 and circUCK2, the correlation between UCK2 and circUCK2 in the development of HCC cells was further explored via rescue experiments. First, MHCC97-H and HCCLM6 cells were introduced with sh-NC + pcDNA, sh-circUCK2 + pcDNA, or sh-circUCK2 + pcDNA-UCK2. As presented in Fig. 5A, the reduced tendency of UCK2 expression induced by circUCK2 knockdown was overturned by UCK2 overexpression. Furthermore, the introduction of pcDNA-UCK2 attenuated circUCK2 interference-caused suppression on cell proliferation (Fig. 5B), colony formation ability (Fig. 5C) and promotion on cell apoptosis (Fig. 5D) in transfected MHCC97-H and HCCLM6 cells. Moreover, the migration and invasion abilities of MHCC97-H and HCCLM6 cells were inhibited by circUCK2 downregulation, whereas these effects were partially alleviated by UCK2 enhancement (Fig. 5E, F). Besides, the increased level of E-cadherin and the decreased levels of N-cadherin and MMP-9 in MHCC97-H and HCCLM6 cells transfected with sh-circUCK2 were all weakened by UCK2 overexpression (Fig. 5G). The efficiency of UCK2 silencing is shown in Figure S1C. UCK2 silencing also attenuated circUCK2 overexpression-induced effects on the proliferation, apoptosis, migration, and invasion of MHCC97-H cells and the protein expression of UCK2, E-cadherin, N-cadherin and MMP-9 (Figure S1D–I). These findings evidenced that circUCK2 modulated HCC cell development in vitro through upregulating UCK2 expression.

### CircUCK2 targeted mir-149-5p to regulate UCK2 expression

**Fig. 6:**
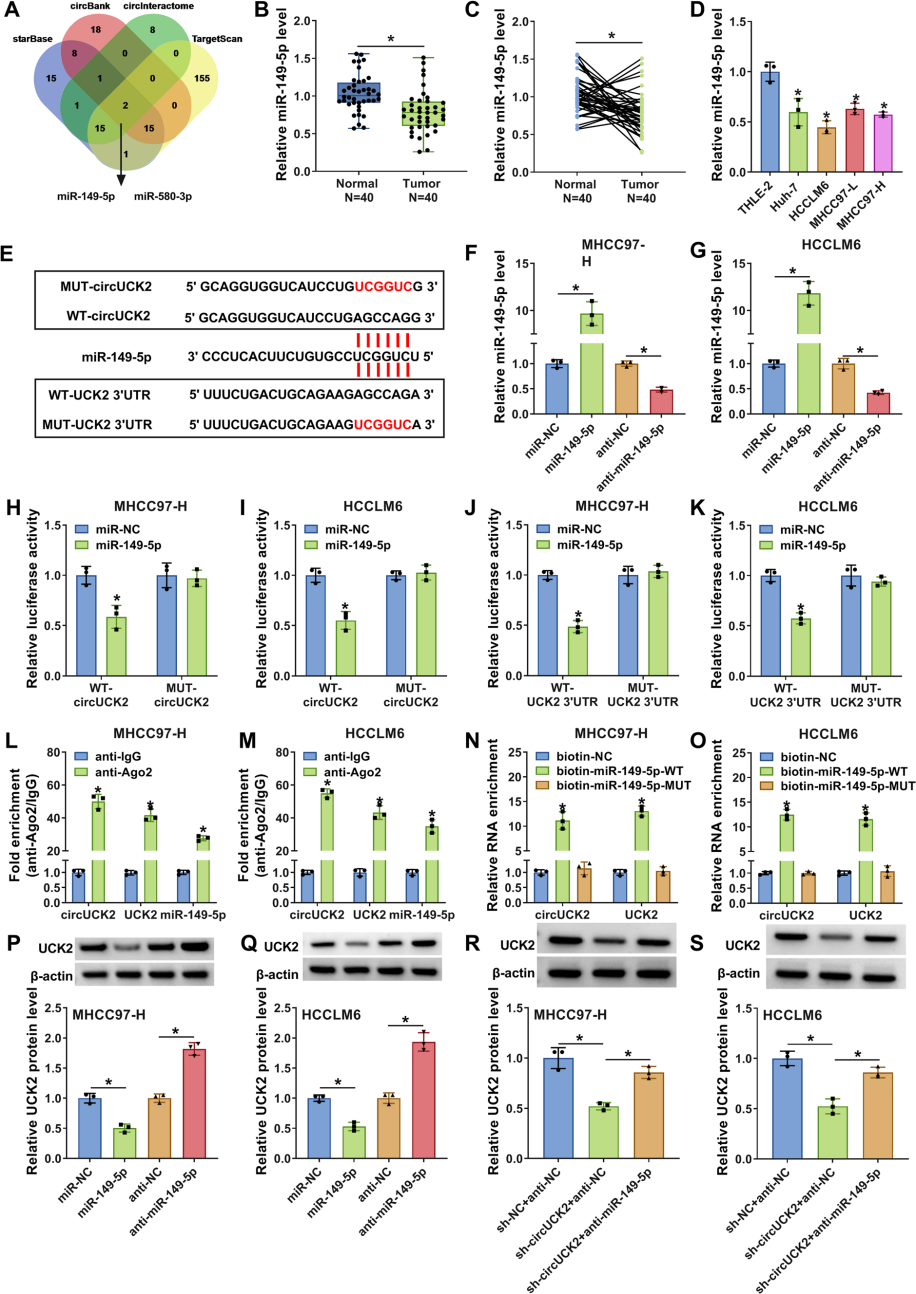
CircUCK2 targeted miR-149-5p to upregulate UCK2 expression. **A** Venn Diagram analysis showed the common miRNAs that binds to UCK2 or circUCK2 predicted by bioinformatics tools, including starBase, circinteractome and circBank and TargetScan. **B**, **C** The expression of miR-149-5p in HCC tissues (*N* = 40) and matched normal tissues (*N* = 40) was detected by qRT-PCR assay. **D** The level of miR-149-5p in THLE-2, Huh7, HCCLM6, MHCC97-L and MHCC97-H cells was estimated by qRT-PCR. **E** Schematic diagram showed the binding sites between miR-149-5p and UCK2 or circUCK2. **F**, **G** The expression of miR-149-5p in MHCC97-H and HCCLM6 cells transfected with miR-NC, miR-149-5p, anti-NC, or anti-miR-149-5p was analyzed with qRT-PCR assay. **H**–**K** The luciferase activity in MHCC97-H and HCCLM6 cells co-transfected with miR-129-5p or miR-NC and WT-circUCK2, MUT-circUCK2, WT-UCK2 3′UTR or MUT-UCK2 3′UTR was determined by dual-luciferase reporter assay. **L**, **M** After RIP assay, the enrichments of circUCK2, UCK2 and miR-149-5p in the immunoprecipitates of MHCC97-H and HCCLM6 cells was determined by qRT-PCR assay. **N**, **O** The RNA enrichments of circUCK2 and UCK2 pulled down by biotin-NC, biotin-miR-149-5p-WT or biotin-miR-149-5p-MUT in MHCC97-H and HCCLM6 cells was examined by qRT-PCR assay. **P**, **Q** The protein expression of UCK2 in MHCC97-H and HCCLM6 cells transfected with miR-NC, miR-149-5p, anti-NC or anti-miR-149-5p was tested by western blot. **R**, **S** The expression of UCK2 in MHCC97-H and HCCLM6 cells transfected with sh-NC + anti-NC, sh-circUCK2 + anti-NC or sh-circUCK2 + anti-miR-149-5p was examined by western blot. *P < 0.05

The circRNA/miRNA/mRNA axis has been reported in HCC [20]. Thereby, the potential miRNAs participating in the progression of HCC through circUCK2/miRNA/UCK2 pathway was explored. Bioinformatics tools including starBase, circinteractome and circBank were used to predict the potential target miRNAs of circUCK2, while bioinformatics tool TargetScan was utilized for the prediction of potential miRNAs that had combinative sites with UCK2. Venn Diagram analysis showed that only two miRNAs, including miR-149-5p and miR-580-3p were included among three tools (Fig. 6A). However, there was no significant change in the expression of miR-580-3p in HCC tissues and cells (not shown in Figures), therefore we selected miR-149-5p for subsequent studies. QRT-PCR analysis presented that the expression of miR-149-5p was markedly declined in HCC tissues (*N* = 40) in comparison with that in matched normal tissues (*N* = 40) (Fig. 6B, C). Consistently, the level of miR-149-5p was also decreased in human HCC cells (Huh-7, HCCLM6, MHCC97-L and MHCC97-H) versus that in normal THLE-2 cells (Fig. 6D).

According to the above predictions, the potential binding sites between miR-149-5p and circUCK2 or UCK2 were presented in Fig. 6E. Then miR-149-5p or anti-miR-149-5p was successfully transfected into MHCC97-H and HCCLM6 cells for the overexpression or knockdown of miR-149-5p (Fig. 6F, G). Dual-luciferase reporter assay proved that miR-149-5p overexpression obviously inhibited the luciferase activities of WT-circUCK2 and WT-UCK2 3′UTR in MHCC97-H and HCCLM6 cells, while there was no change in those of MUT-circUCK2 and MUT-UCK2 3′UTR groups (Fig. 6H, K). Furthermore, RIP assay showed that the enrichments of circUCK2, miR-149-5p and UCK2 were increased in anti-Ago2 immunoprecipitates of MHCC97-H and HCCLM6 cells relative to that in anti-IgG control groups (Fig. 6L, M). Similarly, RNA pull-down assay displayed that biotin-miR-149-5p-WT could pull down more circUCK2 and UCK2 in MHCC97-H and HCCLM6 cells than that in biotin-NC or biotin-miR-149-5p-MUT groups (Fig. 6N, O). These data together confirmed that miR-149-5p could bind to circUCK2 and UCK2. Furthermore, miR-149-5p overexpression resulted in the decreased protein level of UCK2 in both MHCC97-H and HCCLM6 cells, while miR-149-5p silencing caused the opposite effect (Fig. 6P, Q). Besides, the protein abundance of UCK2 was noticeably impaired in circUCK2-silenced cells, which was largely recovered by miR-149-5p knockdown (Fig. 6R, S). All these results provide evidence that circUCK2 could regulate UCK2 expression via sponging miR-149-5p.

### Inhibition of miR-149-5p reversed the effects of circUCK2 knockdown on the malignant progression of HCC cells

**Fig. 7:**
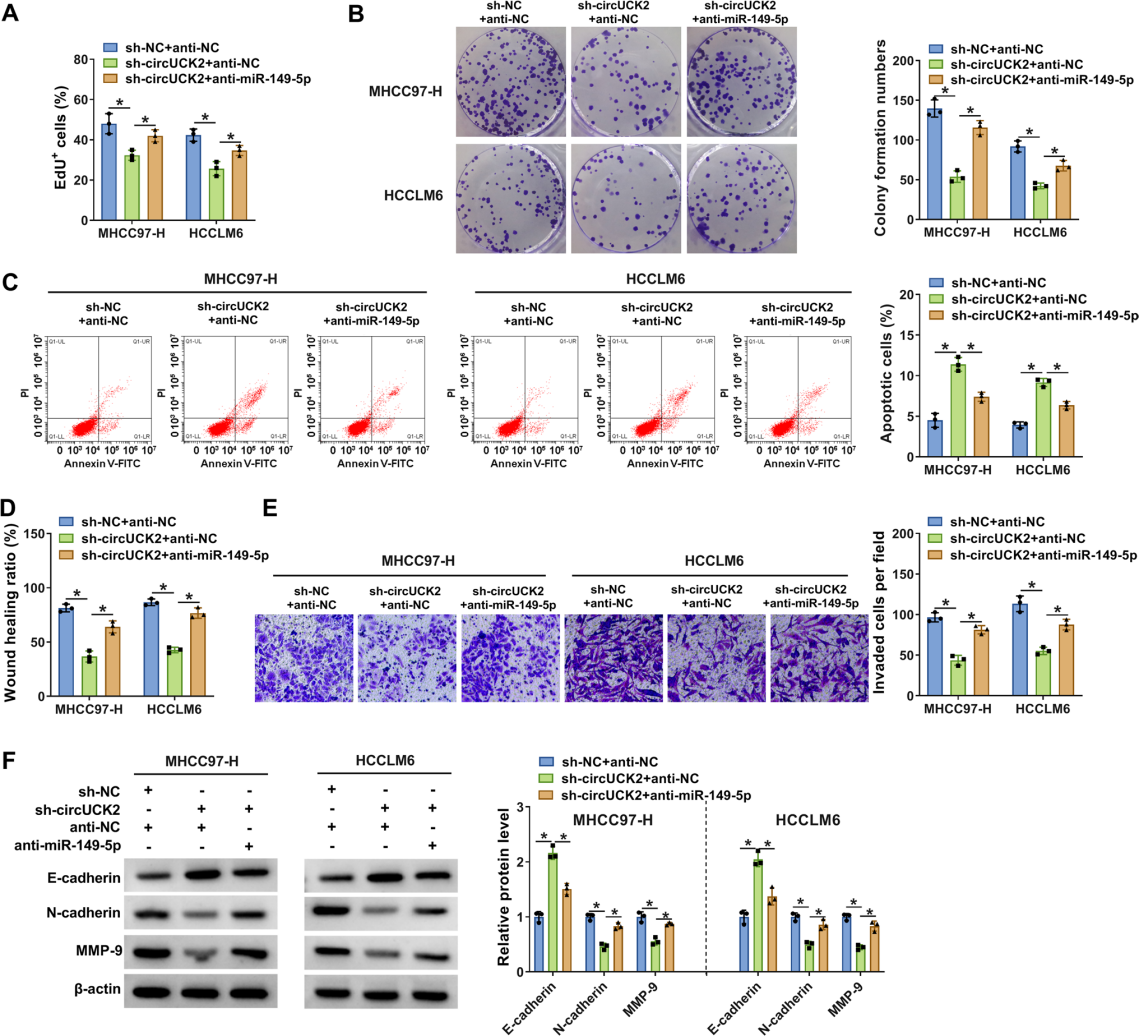
Inhibition of miR-149-5p overturned the effects of circUCK2 knockdown on the progression of HCC cells. MHCC97-H and HCCLM6 cells were transfected with sh-NC + anti-NC, sh-circUCK2 + anti-NC or sh-circUCK2 + anti-miR-149-5p, followed by EdU incorporation assay for cell proliferation (**A**), colony formation assay for colony formation (**B**), flow cytometry for cell apoptosis (**C**), wound healing assay for cell migration (**D**), transwell assay for cell invasion (**E**), and western blot for the expression of E-cadherin, N-cadherin and MMP-9 (F). *P < 0.05

To verify whether miR-149-5p was involved in the regulation of circUCK2 on HCC cell development, MHCC97-H and HCCLM6 cells were transfected with sh-NC + anti-NC, sh-circUCK2 + anti-NC or sh-circUCK2 + anti-miR-149-5p to conduct rescue experiments. Compared with the negative controls, sh-circUCK2 exerted anti-proliferation (Fig. 7A), anti-colony formation (Fig. 7B), pro-apoptosis (Fig. 7C), anti-migration (Fig. 7D) and anti-invasion (Fig. 7E) effects on the malignant phenotypes of MHCC97-H and HCCLM6 cells, which were dramatically mitigated by the introduction of anti-miR-149-5p. Additionally, the enhanced effect of circUCK2 deficiency on E-cadherin expression and decreased effects on N-cadherin and MMP-9 expression in MHCC97-H and HCCLM6 cells were all relieved by miR-149-5p inhibition (Fig. 7F). The efficiency of miR-149-5p overexpresison is shown in Figure S1B. miR-149-5p introduction also attenuated circUCK2 overexpression-induced effects on the proliferation, apoptosis, migration, and invasion of MHCC97-H cells and the protein expression of UCK2, E-cadherin, N-cadherin and MMP-9 (Figure S1D–I). In short, circUCK2 could regulate HCC progression by targeting miR-149-5p.

### CircUCK2 knockdown hindered tumor growth in vivo

**Fig. 8:**
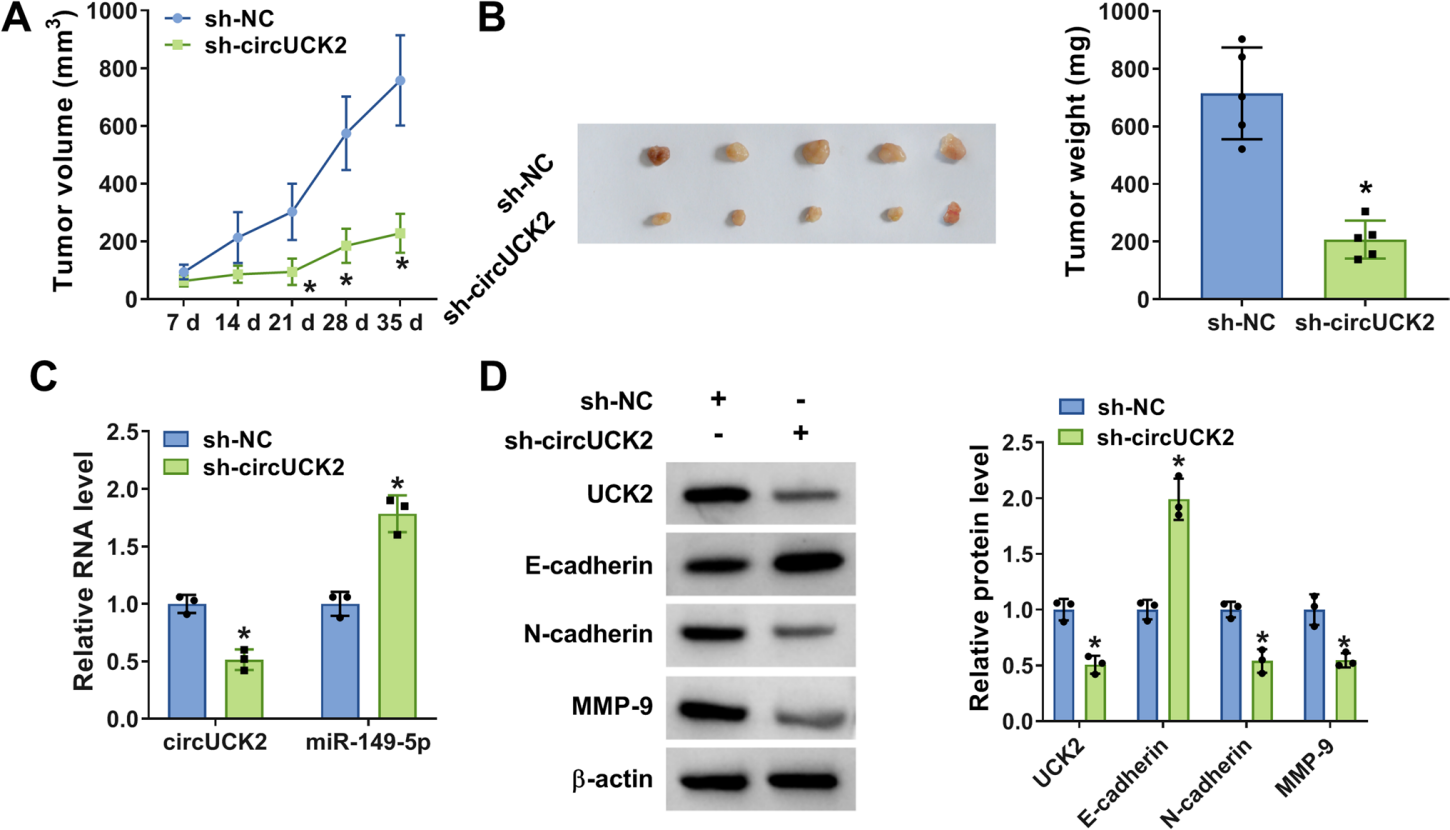
CircUCK2 knockdown inhibited tumor growth in vivo. HCCLM6 cells transduced with sh-circUCK2 or sh-NC were subcutaneously injected into nude mice (*n* = 5/group). **A** Tumor volume was measured every 7 days. **B** The representative images of tumors were presented and tumor weight was examined at day 35 post-injection. **C** The expression of circUCK2 and miR-149-5p in collected tumor tissues from different groups was measured by qRT-PCR. **D** The protein expression levels of UCK2, E-cadherin, N-cadherin and MMP-9 in xenograft tumor tissues were tested by western blot. *P < 0.05

To further confirm the oncogenicity of circUCK2 in HCC cells in vivo, a xenograft tumor model was established by subcutaneous injection of HCCLM6 cells stably transfected with sh-circUCK2 or sh-NC into mice (*n* = 5/group). By contrast with the sh-NC group, tumor volume and weight were prominently restrained in the sh-circUCK2 group (Fig. 8A, B). Furthermore, the levels of circUCK2 and UCK2 were obviously inhibited and miR-149-5p expression was markedly increased in the xenograft tumors derived from sh-circUCK2-transduced cells (Fig. 8C, D). Moreover, the expression of E-cadherin was increased and the expression of N-cadherin and MMP-9 was decreased in xenograft tumor tissues with sh-circUCK2 transfection (Fig. 8D). Collectively, circUCK2 knockdown repressed tumor growth and metastasis in HCC in vivo via regulating miR-149-5p and UCK2.

## Discussion

CircRNAs are intimately linked to the malignant advancement of multifarious cancers by serving as tumor suppressors and oncogenic drivers [21]. Herein, we were the first to confirm the exact function of circUCK2 in HCC progression, and identify the modulatory network of circUCK2/miR-149-5p/UCK2 pathway.

Several circRNAs have been uncovered to serve as crucial mediators in tumor progression and potential diagnostic biomarkers in HCC [8]. CircEPB41L2 exerted a carcinogenic role in HCC through interacting directly with miR-590-5p [22]. CircTP63 was conspicuously enhanced in HCC tissues and cells and accelerated HCC progression via miR-155-5p/ZBTB18 regulatory network [23]. The analysis of GSE94508 dataset showed circUCK2, a splicing form of UCK2, is highly expressed in HCC tissues. Nevertheless, the function of circUCK2 in HCC remains unknown. CircUCK2 were able to mitigate cellular apoptosis and ameliorate neuronal injury after cerebral ischemia-reperfusion injury [24]. And Xiang et al. manifested that circUCK2 knockdown facilitated the invasion and proliferation in prostate cancer cells via sponging miRNA-767-5p [25]. Here, we confirmed the significant overexpression of circUCK2 in HCC tissues and serum relative to normal samples. ROC curve analysis also indicated that circUCK2 might have diagnostic value for HCC patients. Moreover, in vitro experiments suggested circUCK2 was upregulated in HCC cells. Besides, for the first time, we revealed circUCK2 deficiency could impede the proliferation and promoted apoptosis in HCC cells and restrained tumor growth in vivo through loss-of-function experiments. EMT has been attested to be closely concerned with tumor progression and cell migration and invasion [26]. Here, sh-circUCK2 introduction suppressed cell migration and invasion, confirming that circUCK2 knockdown inhibited HCC cell motility by blocking the EMT process. But circUCK2 overexpression displayed the opposite effects. However, the expression patterns and function of circUCK2 in HCC were contrary to the results in prostate cancer, which might due to the different regulatory mechanisms in different type of diseases and the tissue specificity. Collectively, all these data indicated that circUCK2 knockdown exerted tumor-suppressive function in the malignant behaviors of HCC.

Additionally, UCK2, as a precursor linear RNA molecule of circUCK2, was also investigated in this study. UCK2 is reported to be a carcinogenic factor in many malignancies, including lung cancer [27], pancreatic cancer [28] and breast cancer [29]. For instance, UCK2 was upregulated in breast cancer, and elevated UCK2 expression was related to poor prognosis [29]. UCK2 knockdown could restrain lung cancer cell growth [27]. Furthermore, UCK2 was upregulated in HCC and associated with HCC malignant behaviors and poor prognosis [17, 30, 31]. Moreover, the elevated expression of UCK2 is found in HCC tissues, and it is related to a shorter overall survival of HCC patients on the grounds of GEPIA database. Conformably, this study affirmed that UCK2 was overexpressed in HCC tissues and cells. Likewise, IHC assay also verified the elevated expression of UCK2 in HCC tissues. Meanwhile, UCK2 abundance was positively regulated by circUCK2, suggesting that UCK2 was likely associated with the circUCK2-mediated regulatory axis in the pathogenesis of HCC. Interestingly, overexpression of UCK2 effectively mitigated the impacts of circUCK2 knockdown, and UCK2 silencing attenuated circUCK2 overexpression-induced effects. Therefore, we speculated circUCK2 could affect HCC progression by upregulating UCK2 expression.

CircRNA-mediated circRNA/miRNA/mRNA interaction network is an important regulatory mechanism in HCC pathogenesis [32, 33]. For instance, Wang et al. have proposed a regulatory network of circ_0021093/miR-432/Annexin A2 (ANXA2) in HCC development [34]. To explore the circUCK2/miRNA/UCK2 regulatory network in HCC, the common miRNAs that bind to circUCK2 and UCK2 were explored. Through the prediction of bioinformatics tools and the identification of qRT-PCR, miR-149-5p was chosen as our subsequent research object. As previously reported, miR-149-5p was downregulated in cervical cancer and inhibited proliferation and metastasis of cervical cancer cells [35]. Meanwhile, circ-FOXM1 deficiency suppressed lung cancer progression by blocking the expression of miR-149-5p [36]. Also, miR-149-5p was downregulated in HCC tissues and could repressed HCC cell growth through interaction with lncRNA NEAT1 [16, 37]. Expectedly, our data revealed low miR-149-5p expression in HCC tissues and cells was downregulated. Then, the reciprocities between circUCK2 or UCK2 and miR-149-5p were furtherly validated. Concurrently, the level of UCK2 was verified to be reversely regulated by miR-149-5p, and circUCK2 could positively modulate UCK2 level through sponging miR-149-5p. Moreover, the rescue experiments indicated the repressive affects of circUCK2 depletion on HCC cel processes were effectually abated by miR-149-5p silencing. MiR-149-5p introduction also attenuated circUCK2 overexpression-induced effects. The obove data implied the modulatory function of circUCK2 in HCC progression via controlling miR-149-5p.

However, HCC cell lines and xenograft mouse assay can provide useful information for studying the occurrence and progression of HCC, but they cannot fully represent the actual situation of actual patients. This is because HCC is a highly heterogeneous tumor, and its development is influenced by many factors, including genetic factors, environmental factors, and tumor microenvironment. Therefore, a single cell line or animal model may not fully simulate the disease process of patients. In addition, no therapeutic experiments were conducted in this study.

**Fig. 9:**
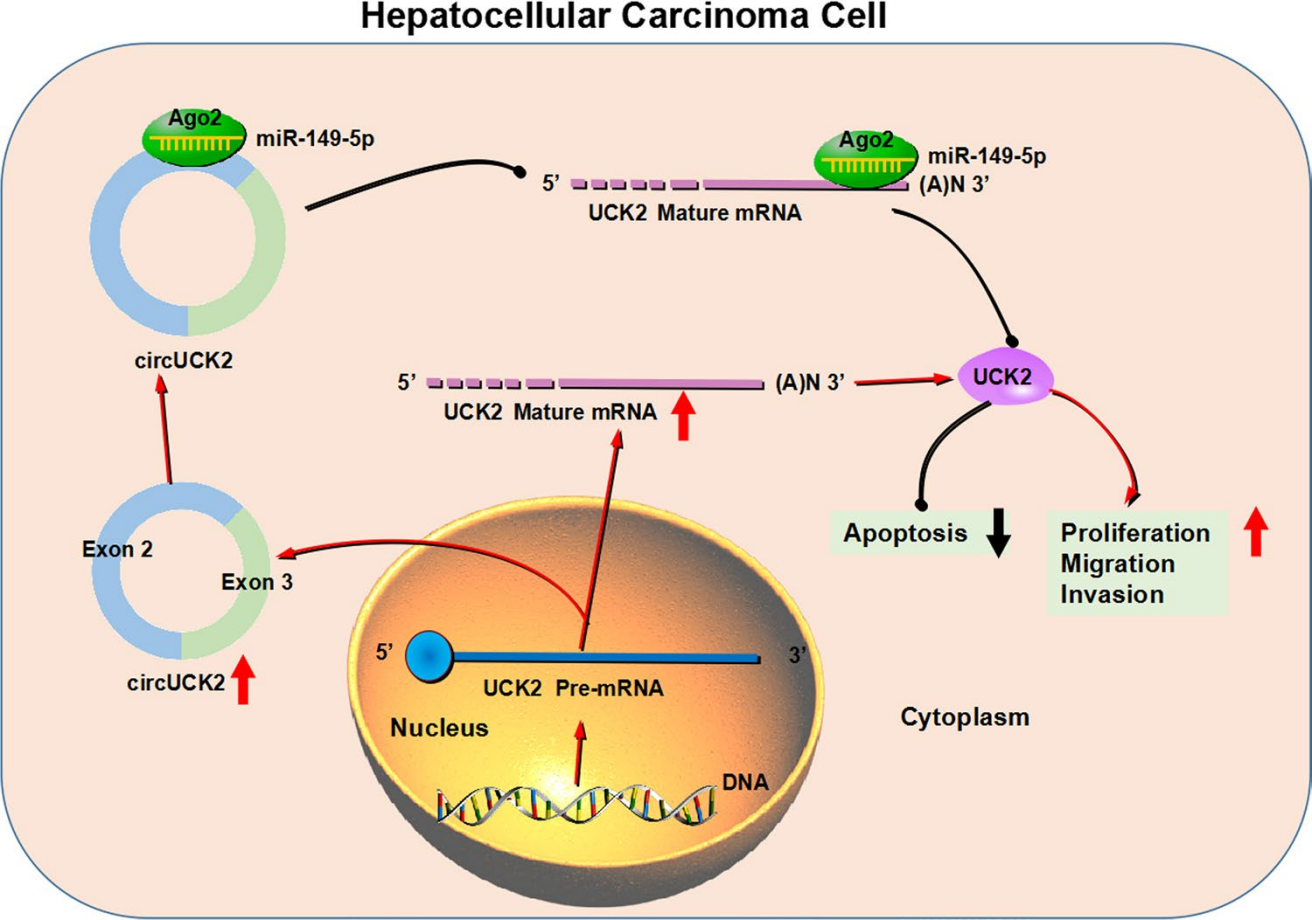
The mechanism responsible for the regulation of circUCK2 in HCC cell malignant phenotypes. HCC cells showed a high circUCK2 expression, and the elevated circUCK2 expression promoted UCK2 expression in a miR-149-5p-dependent manner, finally promoting cell proliferation, migration and invasion and repressing cell apoptosis

Summarily, the study suggested that circUCK2 expression was upregulated in HCC cells. Increased circUCK2 expression induced UCK2 production by segregating miR-149-5p, further promoting the proliferation, migration and invasion and inhibiting the apoptosis of HCC cells (Fig. 9). Our findings offered a feasible mechanism for the participation of circUCK2 in HCC progression and provided evidence for targeting circUCK2 as a novel therapeutic approach for HCC patients.

### Supplementary Information

Below is the link to the electronic supplementary material.

Supplementary file 1 (DOCX 494 KB)

## Data Availability

All data needed to evaluate the conclusions of this paper are presented in the paper and/or the supplementary materials.
