# Supplementary material for: CircUCK2 promotes hepatocellular carcinoma development by upregulating UCK2 in a mir-149-5p-dependent manner
Source: Discov Oncol. 2024 Jan 20;15:14. doi: 10.1007/s12672-024-00863-y (PMC10799813; doi:10.1007/s12672-024-00863-y)
Supplement: Supplementary file 1 — (DOCX 494 KB) [file 12672_2024_863_MOESM1_ESM.docx]

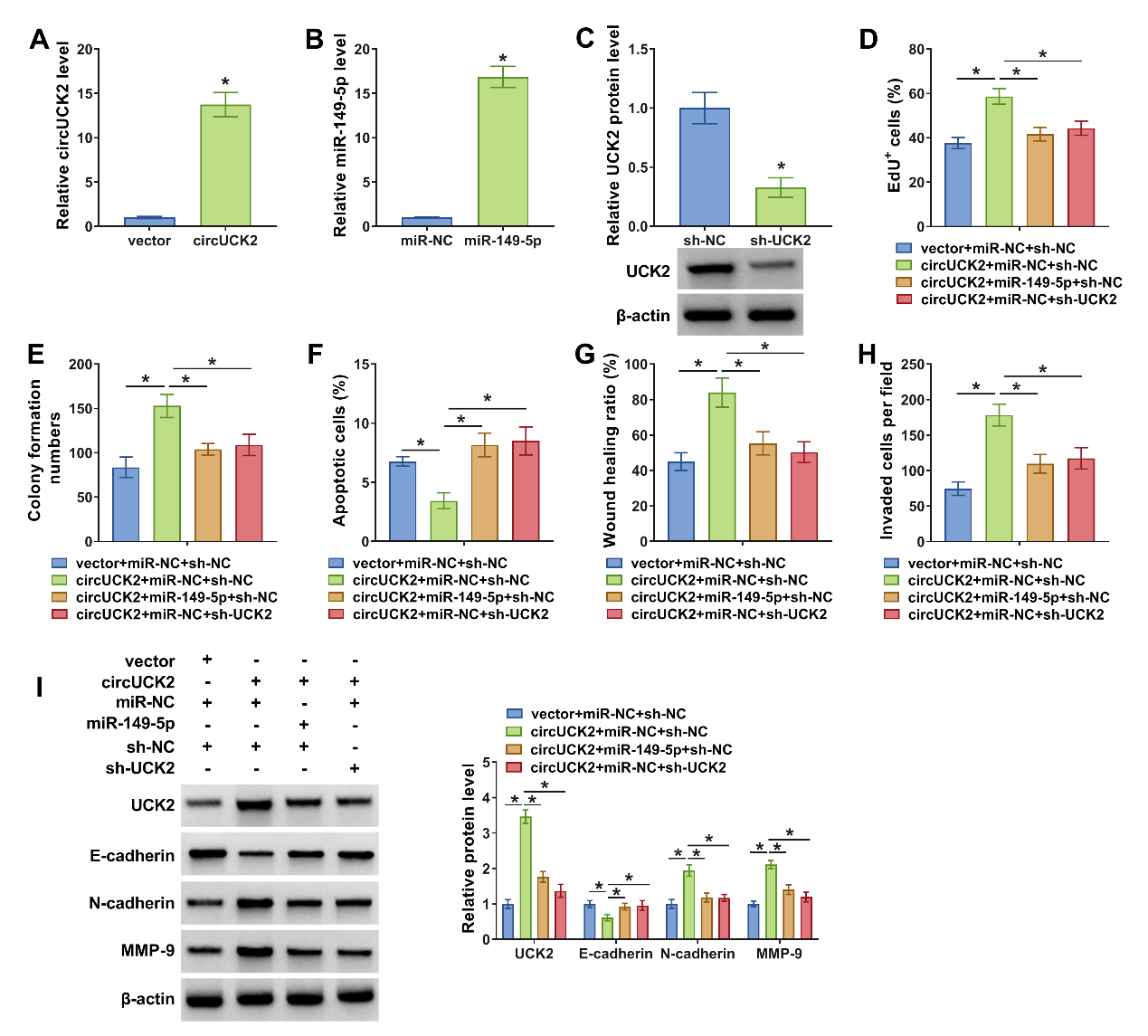


**Figure S1 MiR-149-5p introduction or UCK2 silencing attenuated the promoting effects of circUCK2 overexpression on HCC cell malignancy.** (A and B) The efficiency of circUCK2 and miR-149-5p overexpression was analyzed by qRT-PCR. (C) UCK2 protein expression was detected by western blot after tranfection with sh-UCK2 or sh-NC. (D-I) MHCC97-H cells were divided into vector+miR-NC+sh-NC group, circUCK2+miR-NC+sh-NC group, circUCK2+miR-149-5p+sh-NC group, and circUCK2+miR-NC+sh-UCK2 group. Cell proliferation (D), colony formation (E), apoptosis (F), migration (G) and invasion (H) in transfected cells were examined by EdU incorporation assay, colony formation assay, flow cytometry, wound healing assay and transwell assay, respectively. (I) The protein levels of E-cadherin, N-cadherin and MMP-9 were measured by western blot. **P* < 0.05.


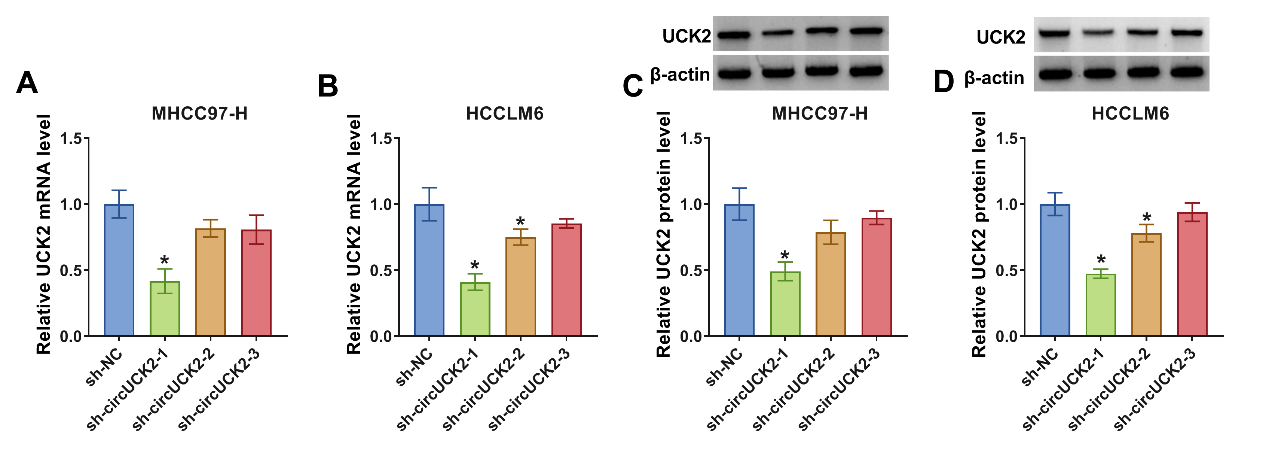


**Figure S2 The effect of Sh-circUCK2 on UCK2 expression.** MHCC97-H and HCCLM6 cells were transfected with sh-circUCK2-1, sh-circUCK2-2 or sh-circUCK2-3. (A and B) The mRNA expression of UCK2 was detected by qRT-PCR. (C and D) The protein expression of UCK2 was detected by western blot. **P* < 0.05.
